# Repeated Wingate sprints is a feasible high-quality training strategy in moderate hypoxia

**DOI:** 10.1371/journal.pone.0242439

**Published:** 2020-11-13

**Authors:** Andreas Breenfeldt Andersen, Jacob Bejder, Thomas Bonne, Niels Vidiendal Olsen, Nikolai Nordsborg

**Affiliations:** 1 Department of Nutrition, Exercise and Sports, University of Copenhagen, Copenhagen, Denmark; 2 Department of Neuroscience and Pharmacology, University of Copenhagen, Copenhagen, Denmark; 3 Department of Neuroanesteshia, The Neuroscience Center, Copenhagen University Hospital (Rigshospitalet), Copenhagen, Denmark; University of Bourgogne France Comté, FRANCE

## Abstract

Sprint-interval training (SIT) is efficient at improving maximal aerobic capacity and anaerobic fitness at sea-level and may be a feasible training strategy at altitude. Here, it was evaluated if SIT intensity can be maintained in mild to moderate hypoxia. It was hypothesized that 6 x 30 s Wingate sprint performance with 2 min active rest between sprints can be performed in hypoxic conditions corresponding to ~3,000 m of altitude without reducing mean power output (MPO). In a single-blinded, randomized crossover design, ten highly-trained male endurance athletes with a maximal oxygen uptake ($\dot{V}$O_2max_) of 68 ± 5 mL O_2_ × min^-1^ × kg^-1^ completed 6 x 30 s all-out Wingate cycling sprints separated by two-minute active recovery on four separate days in a hypobaric chamber. The ambient pressure within the chamber on each experimental day was 772 mmHg (~0 m), 679 mmHg (~915 m), 585 mmHg (~ 2,150 m), and 522 mmHg (~3,050 m), respectively. MPO was not different at sea-level and up to ~2,150 m (~1% and ~3% non-significant decrements at ~915 and ~2,150 m, respectively), whereas MPO was ~5% lower (P<0.05) at ~3,050 m. Temporal differences between altitudes was not different for peak power output (PPO), despite a main effect of altitude. In conclusion, repeated Wingate exercise can be completed by highly-trained athletes at altitudes up to ~2,150 m without compromising MPO or PPO. In contrast, MPO was compromised in hypobaric hypoxia corresponding to ~3,050 m. Thus, SIT may be an efficient strategy for athletes sojourning to moderate altitude and aiming to maintain training quality.

## Introduction

Traveling to terrestrial altitude training-camps to increase sea-level performance is a widespread strategy employed by elite athletes [1]. To allow for proper acclimatization due to a reduced aerobic capacity at altitude, training volumes are recommended to be reduced to avoid overtraining [2]. One potential strategy to improve performance or avoiding a detraining effect during the reduced training volume period is to increase the intensity. Inded, the combinatiation of hypoxic exposure and all-out sprint efforts has been previously suggested [3, 4]. Thus, exploration of training regimes allowing training quality to be upheld while residing at terrestrial altitude, i.e. ‘live high–train high’, is warranted.

In normoxia, various protocols of high-intensity interval training have continuously proven efficient for improving aerobic and anaerobic exercise capacity in both healthy [5, 6] and trained [7, 8] individuals. Specifically, sprint-interval training (SIT) performed as four to six ~30 s Wingate sprints performed in cycle-ergometers interspersed with 2–4 min of passive recovery [9] is a highly efficient training paradigm to improve maximal oxygen uptake ($\dot{V}$O_2max_) and anaerobic performance both in healthy and untrained individuals [10–12] as well as trained individuals [13, 14]. Thus, SIT may represent a feasible way to maintain training intensity at altitude, and a number of training models combining altitude training and SIT have previously been investigated [3, 4, 8, 15–18]. Further, it has been reported that short-sprint (10 to 30 s) performance is maintained at altitudes of 3,300–5,800 m, whereas severe hypoxia with a fraction of inspired O_2_ (F_i_O_2_) of 12–13.3% have been reported to affect overall repeated sprint performance lasting less than 10 s with 20–30 s rest [15, 19–24]. Interestingly, 4 x 30 s all-out efforts separated by 4 min of active rest can be completed by subjects with a history of cycle endurance training at an F_i_O_2_ of 16.4% (~2,000 m) as well as 13.6% (to ~3,500 m) without a significant reduction of exercise intensity [25]. Since repeated SIT (8 x 30 s sprint training with 1.5 min recovery) seems more beneficial than 6 s sprint training (15 x 6 s sprints with 1 min recovery) [26], one may argue that highly-trained athletes performing repeated 30 s all-out sprinting in hypoxia may constitute an efficient training methodology [18]. Furthermore, it should be explored if more than four 30 s all-out bouts can be completed in various degrees of hypoxia in order to maximize the training effect for athletes [11, 25]. Finally, it may be feasible to reduce the resting period between sprints compared to previous investigations [25].

The present study is the first to investigate if well-characterized highly-trained endurance athletes are able to complete a severe SIT training session of more than four 30 s Wingate sprints at various degrees of hypoxia corresponding to ~1.000 m, ~2,000 m, and ~3,000 m. It was hypothesized that 6 x 30 s Wingate sprint performance with 2 min active recovery between sprints can be performed in hypoxic conditions corresponding to ~3,000 m of altitude without reducing mean power output (MPO).

## Methods

### Subjects

Ten non-smoking, White male athletes who had been regularly involved in high-level endurance training (predominantly cycling, triathlon and rowing) participated in the study. Their average age, height, weight and relative $\dot{\text{V}}$O_2max_ ± standard deviation (SD) were 28 ± 7 yrs, 181 ± 5 cm, 74 ± 6 kg and 68 ± 5 mL × min^-1^ × kg^-1^. To prevent bias from previous altitude acclimatization, no subjects who had traveled to altitudes higher than 1,000 m within two months before the study were included. All subjects were low-altitude residents and had not been donating blood for at least three months prior to the study. The subjects were instructed not to travel to high altitudes or donate blood for other purposes during the study period. All subjects were informed both orally and in writing of potential risks and discomforts associated with participation before a written consent was obtained from each subject. The study was approved by the Capital Region’s Committee on Health Research Ethics, Copenhagen, Denmark (H-17012101) and performed in accordance with the guidelines in the Declaration of Helsinki.

### Design

The study was conducted using a single-blinded, randomized cross-over design in which all subjects had to complete 6 repeated Wingate cycling sprints on four separate days in a hypobaric chamber. The six all-out Wingate sprints were conducted in different hypoxic environments with a barometric pressure of 772 mmHg (~0 m), 679 mmHg (~915 m), 585 mmHg (~2,150 m), and 522 mmHg (~3,050 m). Participants were blinded to the prevailing barometric pressure. All participants completed a sea-level $\dot{\text{V}}$O_2max_ test before initiation of the study. The exercise protocol on the experimental days consisted of 10-min warm-up (~100 W) in the hypoxic chamber followed by 5-min recovery. Test procedures are described in detail in the “*Measurements*” section. Finger capillary blood samples were collected immediately after each sprint for determination of blood metabolites. At least 24 hours separated two experimental days, and no more than 14 days separated two experimental days. Subjects were asked to refrain from any strenuous exercise 24 hours before the tests, and not to ingest any ergogenic substances such as caffeine as well as keep their diet similar in the 24 hours preceding the test. All tests were conducted between 10 a.m. and 6 p.m. If data points where missing, these were either inter- or extrapolated; i) within subject, within treatment (e.g. individual data points for a certain sprint within one altitude), or ii) within subject, between treatments (e.g. individual data points for a certain sprint between altitudes), or iii) using a conservative “carry-forward” effect (e.g. sixth sprint equivalent to fifth sprint within one altitude).

### Measurements

#### $\dot{\text{V}}$O_2max_

The $\dot{V}$O_2max_ test was performed on an electronically braked cycling ergometer (Monark 839e, Varberg, Sweden). F_i_O_2_, fraction of inspired CO_2_ (F_i_CO_2_)_,_ fraction of expired O_2_ (F_e_O_2_), fraction of expired CO_2_ (F_e_CO_2_), and pulmonary ventilation (VE) was recorded breath by breath by an automated gas analyzer and ventilation measurement system (Quark, Cosmed, Rome, Italy). Gas analyzers, the flowmeter of the applied spirometer, and the cycling ergometer were calibrated at sea-level according to manufactures guidelines prior to each test. The exercise protocol consisted of 6 min at 90 W, 6 min at 150 W followed by increments of 25 W × min^−1^ until voluntary exhaustion. Subjects were verbally encouraged during the test to perform to exhaustion. Breath-by-breath values were collected and averaged into 5 s averages and $\dot{\text{V}}$O_2max_ was defined as the highest average value over 30 s and a respiratory exchange ratio above 1.15 [27].

#### Repeated Wingate sprint protocol

The repeated Wingate sprints were performed on an electronically braked cycling ergometer (Monark, Varberg, Sweden), which was modified to allow instant application of braking resistance. The test was initiated by subjects reaching >100 rpm during unloaded pedaling and subsequent instant application of 0.095 × kg body mass^-1^ braking resistance. The subjects were encouraged to pedal as fast as possible during the following 30 s. Following the 30 s Wingate sprints, the subjects actively rested for 2 min at ~60 rpm with a load of 1.0 kg corresponding to ~60 W. In the final seconds of the rest period the subjects reached >100 rpm, and the 30 s Wingate sprint was repeated. In total, the subjects performed 6 × 30 s Wingate sprints each separated by 2 min. The highest and lowest power output during any sprint period was automatically recorded and subsequently denoted minimum and peak power output (PPO), respectively. MPO was calculated taking the average value during each sprint. Power variables were blinded for all subjects. As during the $\dot{\text{V}}$O_2max_ protocol, all subjects wore a facemask during each sprint for collection of pulmonary data, which is reported as average values during each sprint. Fatigue index (FI%) was calculated using the following formula: “Fatigue index = minimum power / peak power × 100”. The coefficient of variation of the sprints was calculated using the following formula: CV = (σ /$\overset{-}{\text{X}}$) / √2 where (σ) is the SD of the differences between first sprint at sea level and 1000 m., and ($\overset{-}{\text{X}}$) is the grand mean.

#### Blood samples

Capillary blood samples were collected from a fingertip using 95 μl pre-heparinized tubes (Clinitubes; Radiometer, Brønshøj, Denmark) which were stored on ice until completion of all six sprints. Samples were analyzed using an ABL 800 blood gas analyzer (Radiometer, Brønshøj, Denmark) for blood lactate concentration ([La^-^]), pH, glucose concentration ([glucose]), plasma sodium concentration ([Na^+^]), and plasma potassium concentration ([K^+^]).

### Statistics

All statistics were performed using the IBM SPSS statistical software package v25 (IBM Corp, New York, NY, USA). A mixed model [28] was used to analyze changes in performance variables during the six sprints in different simulated altitude. Fixed factors were sprint (time), altitude (treatment), and sprint × altitude. Repeated measures and random effects were identified by subject. Differences were considered significant if P<0.05. If a significant main effect existed, a post hoc analysis was performed using a Sidak-adjusted pairwise comparison. In addition, effect size was calculated for power output variables, which was then interpreted according to Cohen’s conventional criteria [29] with <0.2, 0.2–0.3, 0.5, and 0.8 representing trivial, small, medium, and large effect sizes, respectively.

A subsequent analysis was performed to investigate whether the variables significantly changed in correlation with increasing altitude. This correlation analysis was performed using a general linear model to calculate the correlation coefficient within subjects by multiple regression [30]. Pearson’s correlation coefficient (r) was converted to a coefficient of determination (r^2^) and used as a measure of the proportion of the variance in the sprint performance that is predictable from the varying altitude. The coefficient of determination was interpreted using a scale of magnitudes (http://www.sportsci.org) [31]. However, as the scale is based on Pearson’s correlation coefficient, the scale was converted to values corresponding to the coefficient of determination. Thus, r^2^ < 0.01 is interpreted as trivial, 0.01–0.09 as small, 0.09–0.25 as moderate, 0.25–0.49 as strong, 0.49–0.81 as very strong and > 0.81 as nearly perfect. Values are presented as means ± standard deviation (SD).

## Results

All 10 subjects completed all six sprints in each simulated altitude. The coefficient of variance was 2.5% for MPO. In regard to inter- and extrapolation, 1.6% and 2.1% data points for MPO and PPO were inter- or extrapolated. All other variables ranged from 2.5–13% with [Na^+^] being the most inter- or extrapolated variable.

### Power output

**Fig 1:**
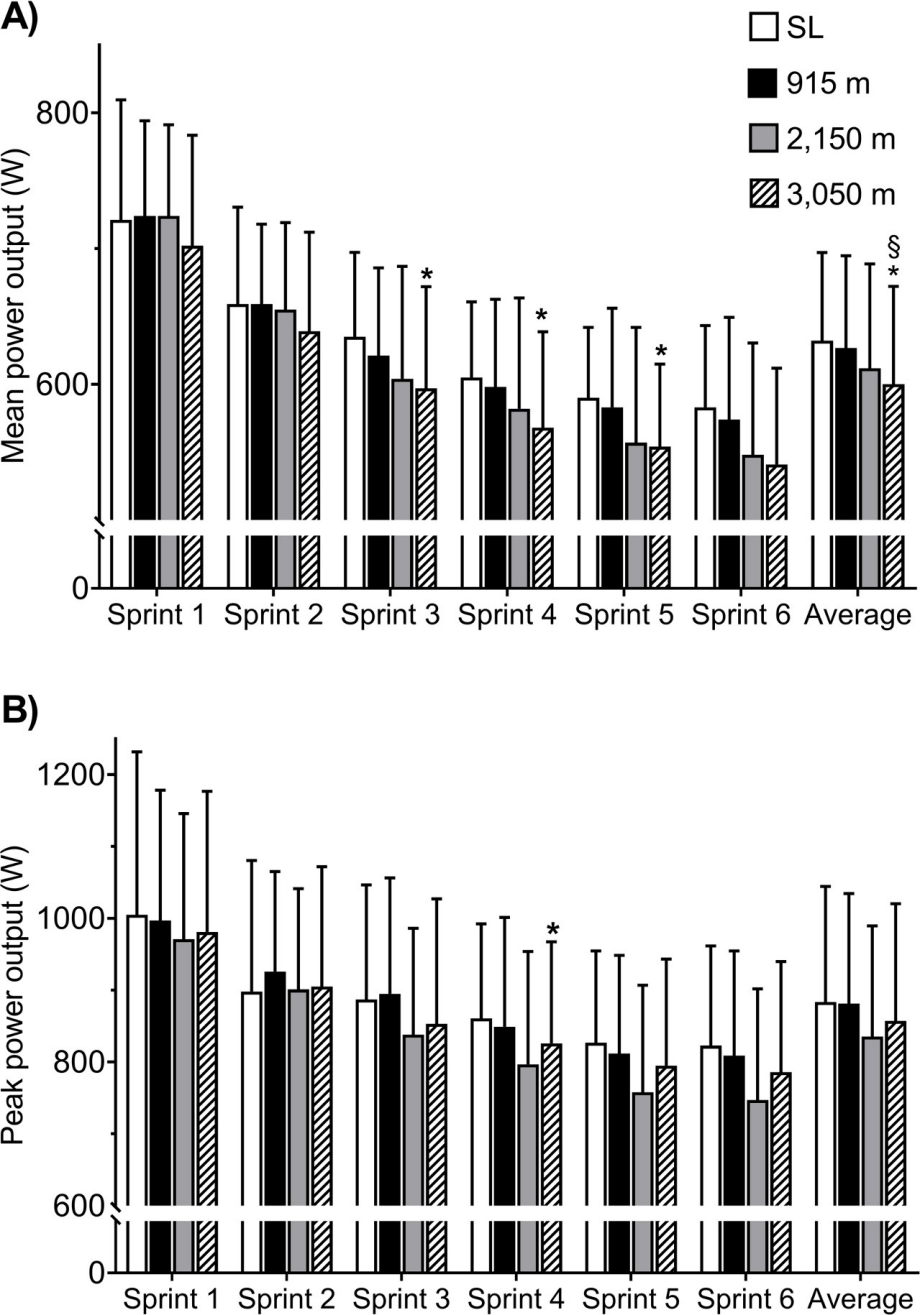
(A) Illustration of the mean power output (MPO) and (B) peak power output (PPO) in watt (W) of each consecutive sprint and average of all six sprints at each altitude. SL: sea-level. * Different from sea-level (P<0.05), § different from 1,000 m (P<0.05).

An overall effect of altitude on MPO was evident (P = 0.004), whereas no sprint × altitude effect was evident (P = 0.46). When comparing sprint 1 and 6 at each altitude, MPO decreased by 19, 21, 24 and 23% at sea-level, 915 m, 2,150 m, and 3,050 m, respectively (P<0.001). As illustrated on Fig 1A, the average MPO for all six sprints at 3,050 m was lower than sea-level by 5% (P = 0.02, effect size -0.28) and 915 m by 4.2% (P = 0.04, effect size -0.23). In the first two sprints, no correlation between MPO and altitude existed. In contrast, MPO exhibited a “strong” negative correlation to altitude for sprint 3–6 (P = 0.005).

A main effect of altitude on PPO was evident whereas no effect of sprint × altitude was found (P<0.001 and P = 0.96, respectively). The reduction of PPO from sprint 1 to 6 was 18, 19, 23, and 20% at sea level, 915 m, 2,150 m and 3,050 m, respectively (P = 0.007). No difference was detectable when comparing the average of sprint 1 to 6 PPO between altitudes (P = 0.25, Fig 1B, effect size = -0.02, -0.31, and -0.19 for 915, 2,150, and 3,050 m, respectively, when compared to sea-level). Moreover, no correlation between PPO and altitude was detectable (Table 1). A main effect for both sprint and altitude was observed for FI% (P<0.001). No interaction of sprint × altitude was found (P = 0.989).

**Table 1:** Correlation between variables and sprints in hypoxic exposure.

	X-variable												
**Y-variable**		**Sprint 1**		**Sprint 2**		**Sprint 3**		**Sprint 4**		**Sprint 5**		**Sprint 6**	
		***r***^***2***^	***r [95% CI]***	***r***^***2***^	***r [95% CI]***	***r***^***2***^	***r [95% CI]***	***r***^***2***^	***r [95% CI]***	***r***^***2***^	***r [95% CI]***	***r***^***2***^	***r [95% CI]***
	**MPO (W)**	0.07	0.26 [0.10; 0.43]	0.10(*)	0.32 [0.15; 0.49]	0.30**	0.54 [0.40; 0.69]	0.32**	0.57 [0.43; 0.71]	0.28**	0.53 [0.38; 0.68]	0.24**	0.49 [0.34; 0.85]
	**PPO (W)**	0.05	0.22 [0.06; 0.38]	0.00	0.01 [0.00; 0.06]	0.12	0.35 [0.18; 0.52]	0.17	0.41 [0.25; 0.58]	0.13	0.36 [0.19; 0.52]	0.13	0.36 [0.19; 0.52]
	**Lactate (mmol × L**^**-1**^**)**	0.05	0.21 [0.06; 0.37]	0.31**	0.56 [0.42; 0.70]	0.20**	0.45 [0.26; 0.61]	0.17*	0.41 [0.25; 0.57]	0.08	0.28 [0.12; 0.45]	0.10 (*)	0.32 [0.15; 0.48]
	**pH**	0.27**	0.52 [0.37; 0.67]	0.07	0.27 [0.11; 0.44]	0.00	0.07 [0.00; 0.17]	0.02	0.13 [0.01; 0.27]	0.02	0.14 [0.01; 0.28]	0.03	0.17 [0.02; 0.32]
	**FI (%)**	0.00	0.05 [0.01; 0.14]	0.06	0.25 [0.09; 0.41]	0.08	0.28 [0.11; 0.44]	0.11(*)	0.33 [0.16; 0.49]	0.15*	0.38 [0.22; 0.55]	0.09	0.30 [0.14; 0.47]
	**K**^**+**^ **(mmol × L**^**-1**^**)**	0.02	0.13 [0.00; 0.27]	0.04	0.19 [0.04; 0.34]	0.04	0.21 [0.05; 0.37]	0.00	0.06 [0.00; 0.16]	0.17*	0.41 [0.25; 0.58]	0.15	0.39 [0.22; 0.56]
	**Na**^**+**^ **(mmol × L**^**-1**^**)**	0.03	0.18 [0.03; 0.33]	0.03	0.17 [0.02; 0.31]	0.11(*)	0.33 [0.16; 0.50]	0.11(*)	0.33 [0.17; 0.50]	0.04	0.20 [0.05; 0.36]	0.03	0.17 [0.02; 0.31]
	**Glucose (mmol × L**^**-1**^**)**	0.03	0.17 [0.02; 0.32]	0.10(*)	0.32 [0.15; 0.49]	0.27*	0.52 [0.37; 0.67]	0.17*	0.41 [0.25; 0.57]	0.16*	0.40 [0.23; 0.56]	0.06	0.25 [0.08; 0.41]
	**VE (L×min**^**-1**^**)**	0.15*	0.39 [0.22; 0.55]	0.20*	0.45 [0.29; 0.61]	0.22**	0.47 [0.31; 0.63]	0.09(*)	0.31 [0.14; 0.47]	0.05	0.22 [0.06; 0.38]	0.00	0.05 [0.00; 0.14]
	**VO**_**2**_ **(mL × min**^**-1**^**)**	0.08	0.28 [0.12; 0.45]	0.00	0.02 [0.00; 0.08]	0.01	0.10 [0.00; 0.23]	0.17*	0.41 [0.25; 0.57]	0.18*	0.43 [0.27; 0.59]	0.42**	0.65 [0.53; 0.77]

Coefficient of determination (r^2^) and coefficient of correlation (r) with 95% confidence intervals ([95% CI]) for measured variables and hypoxic exposure in each sprint. PPO: peak power output, FI: Fatigue Index, K^+^: potassium, Na^+^: sodium, VE: pulmonary ventilation, VO_2_: oxygen uptake. Statistical significant r^2^ are:

* P<0.05,

** P<0.01. Brackets () indicates statistical tendency (P < 0.1).

### Blood metabolites and ions

A main effect of altitude existed for [La^-^] (P<0.001), pH (P = 0.003), [glucose] (P = 0.001), [Na^+^] (P = 0.003) and [K^+^] (P = 0.036; see Table 2). No sprint × altitude effect was observed for any of the variables (p-value ranging between P = 0.287 and P = 0.993). When comparing sprint 1 and 6, [La^-^] increased by 62, 63, 57, and 60% at sea-level, 915 m, 2,150 m, and 3,050 m (P<0.001 for all altitudes), respectively. For [La^-^], a “strong” and “moderate” positive correlation existed between sprint 2 (P = 0.001), 3 and 4 (P = 0.01 and P = 0.02, respectively.) and the simulated altitude, respectively (see Table 2). For pH, a positive “strong” correlation with altitude was found at sprint 1 (see Table 2; P = 0.003). As noted in Table 2, [glucose] correlated positively and “strongly” with altitude at sprint 3 (P = 0.03), and “moderately” at sprint 4 and 5 (P = 0.02). No correlation was found for [K^+^] or [Na^+^].

**Table 2:** Blood metabolites, fatigue index, and pulmonary variables.

Lactate (mmol × L^-1^)#	Sprint 1	Sprint 2	Sprint 3	Sprint 4	Sprint 5	Sprint 6
SL	6.8 ± 1.4	11.6 ± 3.8	14.3 ± 4.0	16.2 ± 4.2	17.8 ± 4.4	18.0 ± 4.4
915 m	6.6 ± 3.4	12.9 ± 3.9	15.0 ± 2.7	17.3 ± 3.6	17.0 ± 3.8	17.6 ± 3.1
2,150 m	7.7 ± 2.0	13.4 ± 3.4	15.1 ± 2.6	17.5 ± 3.4	17.3 ± 2.8	17.9 ± 2.9
3,050 m	7.6 ± 1.7	14.3 ± 3.6 (*)	16.5 ± 3.2§	17.9 ± 4.1	19.2 ± 4.1	19.2 ± 4.2
**pH#**	**Sprint 1**	**Sprint 2**	**Sprint 3**	**Sprint 4**	**Sprint 5**	**Sprint 6**
SL	7.31 ± 0.04	7.20 ± 0.08	7.18 ± 0.06	7.13 ± 0.08	7.11 ± 0.08	7.10 ± 0.09
915 m	7.30 ± 0.04	7.22 ± 0.05	7.17 ± 0.05	7.12 ± 0.07	7.11 ± 0.07	7.10 ± 0.08
2,150 m	7.32 ± 0.03	7.22 ± 0.04	7.16 ± 0.05	7.13 ± 0.06	7.11 ± 0.06	7.09 ± 0.06
3,050 m	7.35 ± 0.03 (§)	7.23 ± 0.06	7.18 ± 0.06	7.15 ± 0.08	7.13 ± 0.08	7.13 ± 0.08
**FI (%)#**	**Sprint 1**	**Sprint 2**	**Sprint 3**	**Sprint 4**	**Sprint 5**	**Sprint 6**
SL	50 ± 12	53 ± 12	56 ± 10	58 ± 12	56 ± 11	58 ± 11
915 m	50 ± 11	52 ± 8	57 ± 9	59 ± 10	56 ± 11	57 ± 9
2,150 m	47 ± 9	53 ± 6	55 ± 9	56 ± 11	58 ± 7	58 ± 11
3,050 m	52 ± 13	57 ± 12	63 ± 15	64 ± 12	64 ± 17	67 ± 18
**K**^**+**^ **(mmol × L**^**-1**^**)#**	**Sprint 1**	**Sprint 2**	**Sprint 3**	**Sprint 4**	**Sprint 5**	**Sprint 6**
SL	6.5 ± 0.8	6.4 ± 1.0	6.1 ± 0.8	5.8 ± 1.0	6.1 ± 1.1	6.1 ± 1.0
915 m	6.9 ± 1.7	6.3 ± 0.8	6.0 ± 0.8	6.0 ± 0.6	5.9 ± 0.3	6.0 ± 0.4
2,150 m	6.4 ± 0.8	6.1 ± 0.5	6.1 ± 0.5	6.0 ± 0.4	5.6 ± 0.5	5.7 ± 0.2
3,050 m	6.4 ± 0.8	6.2 ± 0.4	5.8 ± 0.5	5.9 ± 0.7	5.6 ± 0.4	5.7 ± 0.5
**Na**^**+**^ **(mmol × L**^**-1**^**)#**	**Sprint 1**	**Sprint 2**	**Sprint 3**	**Sprint 4**	**Sprint 5**	**Sprint 6**
SL	145.2 ± 2.9	144.6 ± 2.1	145.4 ± 2.4	145.4 ± 2.2	147.2 ± 4.0	147.3 ± 3.4
915 m	144.6 ± 1.8	145.9 ± 2.6	144.7 ± 2.6	146.5 ± 2.0	146.5 ± 3.0	146.4 ± 2.3
2,150 m	143.3 ± 2.2	143.9 ± 2.8	146.0 ± 4.5	145.6 ± 2.4	144.7 ± 4.3	146.0 ± 1.8
3,050 m	144.6 ± 1.9	146.2 ± 1.3†	146.8 ± 1.5	147.0 ± 1.3	146.4 ± 1.6	146.6 ± 1.5
**Glucose (mmol × L**^**-1**^**)#**	**Sprint 1**	**Sprint 2**	**Sprint 3**	**Sprint 4**	**Sprint 5**	**Sprint 6**
SL	5.2 ± 0.7	5.5 ± 0.8	6.0 ± 0.9	6.4 ± 0.8	6.7 ± 1.0	7.0 ± 1.2
915 m	5.0 ± 1.4	5.8 ± 0.7	6.1 ±0.8	6.6 ± 1.0	6.8 ± 1.1	7.2 ± 1.0
2,150 m	5.5 ± 0.7	6.0 ± 0.8	6.4 ± 0.9	6.8 ± 1.0	6.9 ± 1.1	7.2 ± 1.1
3,050 m	5.4 ± 0.6	5.9 ± 0.4	6.6 ± 0.7 (*)	7.0 ± 1.0	7.3 ± 1.1	7.4 ± 1.1
**VE (L × min**^**-1**^**)#**	**Sprint 1**	**Sprint 2**	**Sprint 3**	**Sprint 4**	**Sprint 5**	**Sprint 6**
SL	132 ± 19	158 ± 24	165 ± 25	170 ± 23	164 ± 28	167 ± 21
915 m	138 ± 18	166 ± 22	171 ± 21	170 ± 22	168 ± 22	170 ± 23
2,150 m	140 ± 18	174 ± 13(*)	178 ± 14	174 ± 17	168 ± 19	167 ± 21
3,050 m	144 ± 20	171 ± 27	176 ± 23*	175 ± 23	169 ± 22	169 ± 26
**VO**_**2**_ **(mL × min**^**-1**^**)#**	**Sprint 1**	**Sprint 2**	**Sprint 3**	**Sprint 4**	**Sprint 5**	**Sprint 6**
SL	3113 ± 732	3413 ± 670	3282 ± 615	3450 ± 504	3394 ± 556	3379 ± 475
915 m	3278 ± 424	3580 ± 396	3480 ± 450	3373 ± 465	3241 ± 463	3193 ± 546
2,150 m	3396 ± 503	3540 ± 566	3446 ± 558	3346 ± 581	3179 ± 532	3083 ± 558(*)
3,050 m	3334 ± 697	3405 ± 662	3307 ± 636	3192 ± 654	3104 ± 640	2977 ± 516*

Venous blood values, fatigue index in percentage (FI%), and pulmonary variables collected after each sprint in each simulated altitude. Values are means ± SD. SL: sea-level, VO_2_: oxygen uptake, VE: pulmonary ventilation, K^+^: potassium, Na^+^: sodium. Statistical significance denoted by: # significant main effect, i.e. effect of altitude (P < 0.05),

* significant different from sea-level (P < 0.05),

§ significantly different from 1,000 m. (P < 0.05),

† significantly different (P < 0.05) from 2,000 m. Brackets () indicates statistical tendency (P < 0.1).

### Pulmonary variables

A main effect of altitude was evident for both $\dot{\text{V}}$O_2_ (P = 0.045) and VE (P<0.001; see Table 2) whereas no sprint × altitude effect was found (P = 0.505 and P = 0.710 respectively). When comparing sprint 1 and 6 in each simulated altitude, no statistical significant changes were apparent at sea-level and 915 m for $\dot{\text{V}}$O_2_. In contrast, $\dot{\text{V}}$O_2_ decreased by 10% and 12% in 2,150 m (P = 0.012) and 3,050 m (P = 0.010), respectively. From sprint 1–6, VE increased with 21, 19, 16, and 15% at sea level (P<0.001), 915 m (P<0.001), 2,150 m (P = 0.03), and 3,050 m (P = 0.002), respectively. A “moderate” negative correlation existed between $\dot{\text{V}}$O_2_ and altitude in sprint 4 and 5 (P = 0.02, P = 0.01 respectively; see Table 2), and a “strong” negative correlation was found for sprint 6 (P<0.001). A “moderate”, positive correlation between VE and hypoxic exposure existed in sprint 1–3 (P = 0.03, P = 0.012, P = 0.008 resp.; see Table 2). Pressure of inspired oxygen (PiO_2_) was 148 ± 4 mmHg, 133 ± 1 mmHg, 113 ± 1 mmHg, and 99 ± 1 mmHg at sea-level and in 915 m, 2,150 m, and 3,050 m, respectively.

## Discussion

The present study is the first to investigate whether 6 x 30 s Wingate sprints can be completed by highly-trained endurance athletes in different hypobaric hypoxic environments without compromising exercise intensity as compared to sea-level. The main finding is that MPO of all six all-out efforts was not different at sea-level and hypobaric hypoxia corresponding to ~1,000 m and ~2,000 m of altitude. In contrast, exercise intensity was compromised in hypobaric hypoxia corresponding to ~3,000 m.

The magnitude of acute hypoxia’s detrimental effect on performance is dependent on the type of exercise and the duration [32, 33]. It has previously been demonstrated that a single 30 or 45 s all-out sprint performance is unaffected by severe hypoxia (F_i_O_2_: 10.0–11.3%) corresponding to 4,800–5,800 m [22, 34, 35] most likely because of the high dependence on anaerobic energy production [22]. However, here we demonstrate that as much as six 30 s supramaximal bouts is feasible in moderate hypoxia corresponding to 2,150 m without compromising power. Previous studies investigating 5–6 s sprints in different hypoxic environments (FiO_2_ 12–21%) report that sprint performance only is attenuated in severe hypoxia [15, 21]. Only one other study has investigated the acute effect of different altitudes on repeated 30 s Wingate sprint performance in individuals with a history of endurance cycling training [25], while others have examined various repeated sprint protocols of shorter duration or with no repetitions primarily in team-sports athletes [18, 20, 36–39]. In the study by Kon et al., the participants completed 4 x 30 s all-out sprints with four-minute active recovery in hypoxic environments corresponding to ~2,000 m and 3,500 m [25]. Despite the difference in number of repeated sprints (four vs. six) and recovery between bouts (two min vs. four min.) between our study and the work by Kon et al., a similar gradual decrease in MPO from first to last sprint in the range of ~20–25% is evident in both studies. Nevertheless, in contrast to our findings, Kon et al. reported no differences in MPO when comparing hypoxic exposure corresponding to ~3,500 m with normoxia [25]. Another study report a decrease of ~10–12% in MPO when participants perform 3 x 30 s Wingate tests with 4.5 min of passive recovery (F_i_O_2_ = 14.5%) [36]. A plausible explanation for the ability to sustain exercise intensity is the completion of fewer sprints and longer recovery periods compared to the present study. Indeed, four-minutes of recovery increases total oxygen uptake [40] which allows more complete PCr resynthesis and increases time for muscle lactate and H^+^ efflux as well as reestablishment of ion-homeostasis which are all suggested fatigue factors [41, 42]. Further, it has also been shown that 6 x 30 s Wingate tests with 4 min rest between sprints results in a reactive oxygen species-dependent ryanodine receptor type 1 fragmentation in muscles of recreationally active subjects, but not in elite endurance athletes ($\dot{\text{V}}$O_2max_ = 52 ± 3 mL × min^-1^ × kg^-1^ and 67 ± 2 mL × min^-1^ × kg^-1^, respectively) [43]. Hence, more severe sprint protocols (e.g. more repetitions and/or shorter recovery) may be needed for similar adaptations in elite athletes.

The average of PPO in sprint 1–6 was not different at all simulated altitudes, which is in agreement with previous findings [25, 34, 44]. Moreover, the similar reduction in PPO from sprint 1 to 6 of ~20% between conditions is in agreement with studies using shorter cycle sprint protocols (4-10s sprint with 20–30 seconds recovery) [17, 20, 22, 39, 45, 46] or repeated 4–6 s running sprint protocols [21, 38, 47] reporting a negligible effect of hypoxia. Thus, the decrement in PPO during SIT seems to be unaffected by hypoxic levels corresponding to an altitude of up to ~3,000 m.

Importantly, the current findings demonstrate that the applied high-intensity training protocol is feasible in altitudes up to 2,150 m, whereas 3,050 m is too severe with the chosen protocol. Whether an increased recovery period in 3,050 m can overcome this issue is unknown. In addition, it remains to be elucidated whether more than six sprints can be performed at 2,150 m without compromising intensity. These findings only applies for a similar cohort as the one investigated in the present study, as the responses to hypoxia also is dependent on the training background [48]. Notably, our findings are well above the present studies detection limit. If a post-experiment power analysis is calculated using the standard deviation of the differences obtained from the first sprint at sea-level and the 1,000 m trial, inclusion of 10 subjects allow a detection of a 20.0 W change with alpha = 0.05 and power > 0.8.

While MPO was maintained at 2,150 m, the decrease in MPO at 3,050 m concurrent with a 12% reduction of $\dot{\text{V}}$O_2_ and maintained VE (Table 2) clearly demonstrate that the investigated protocol cannot be completed without a decrease in MPO at ~3,050 m or higher. This is supported by the main effect of altitude on [La^-^] as well as numerically higher [La^-^] in all sprints at 3,050 m as compared to sea-level with significant differences in sprint two and three. Previous studies have reported no effect of altitude on [La^-^] [22, 25], although not all support this finding [19].

In the present study, we observed no differences between altitudes for neither pH, [glucose], [Na+] nor [K+] despite a main effect for all variables and it cannot be excluded that a difference in these variables were too small to be detected. Previously it has been reported that prolonged hypoxic exposure have resulted in an increased muscle buffer capacity [49], while others report no differences in pH or [K+] after a single 30s Wingate sprint conducted in both hypoxia and normoxia after 4 weeks LHTL [50]. An insignificant, numerical increase for [glucose] were evident, which is likely the results of an increased catecholamine release during sprints [51] resulting in increased release of glucose from the liver.

Moreover, linear regression analyses revealed that only 24–32% of the between trial variation in MPO could be related to variation in the degree of hypoxia within sprint 3 to 6 (see Table 1). Additionally, no more than 17–32% of the variation in [La^-^] after sprint 1–6 was explained by the variation in simulated altitude. These observations support that the proposed exercise model largely allows metabolism during SIT to be unaffected by simulated altitude. However, due to the low number of participants in the present study, the correlation analysis should be interpreted with caution.

A limitation in the present study is the low number of participants and the absence of invasive measurements. Previous studies have conducted full-invasive studies in sprint studies, enabling them to investigate different aspects of the results in depth suggesting an additional hypoxia-related metabolic load [22, 23, 39, 43]. In addition, the maximal period between two experimental days was at a length, which could hypothetically influence the results due to for instance training variations. Furthermore, the extrapolation performed as described previously should be noted. Finally, the present study was only conducted as a single-blinded study due to logistical reasons. Future studies should aim for applying a double-blinded design.

These results support that the combination of a moderate hypoxic stress and all-out sprint efforts are feasible as an altitude training modality as previously suggested [3, 4]. Furthermore, repeated sprint peak power output is maintained during a hypoxic exposure up to 3,050 m. This suggest that athletes could benefit from supramaximal training bouts in hypoxia. Future studies should elucidate if the proposed protocol is feasible when repeated in a relevant cohort such as highly-trained athletes. As acclimatization to altitude usually is recommended to last 7–10 days for athletes [52], it could be hypothesized that athletes could take advantage of SIT in this period. This may ensure a strong training stimulus in the beginning of an altitude training camp concurrent with an adaptation to the O_2_-deprived environment but remains unknown.

## Conclusion

In conclusion, a single session of 6 x 30 s Wingate sprinting is a feasible model for high-intensity training at moderate altitude for highly-trained athletes. Our data demonstrate that the exercise intensity of intermittent sprinting can be sustained in hypoxic environments corresponding to 2,150 m, while more severe hypoxia corresponding to ~3,050 m decrease the exercise power outputs.
